# Complex Network of NKT Cell Subsets Controls Immune Homeostasis in Liver and Gut

**DOI:** 10.3389/fimmu.2018.02082

**Published:** 2018-09-11

**Authors:** Idania Marrero, Igor Maricic, Ariel E. Feldstein, Rohit Loomba, Bernd Schnabl, Jesus Rivera-Nieves, Lars Eckmann, Vipin Kumar

**Affiliations:** ^1^Laboratory of Immune Regulation, University of California, San Diego, La Jolla, CA, United States; ^2^Division of Gastroenterology and Hepatology, Department of Medicine, University of California, San Diego, La Jolla, CA, United States; ^3^Department of Pediatrics, University of California, San Diego, La Jolla, CA, United States

**Keywords:** CD1d, lipids, hepatitis, microbiota, epithelium

## Abstract

The liver-gut immune axis is enriched in several innate immune cells, including innate-like unconventional and adaptive T cells that are thought to be involved in the maintenance of tolerance to gut-derived antigens and, at the same time, enable effective immunity against microbes. Two subsets of lipid-reactive CD1d-restricted natural killer T (NKT) cells, invariant NKT (iNKT) and type II NKT cells present in both mice and humans. NKT cells play an important role in regulation of inflammation in the liver and gut due to their innate-like properties of rapid secretion of a myriad of pro-inflammatory and anti-inflammatory cytokines and their ability to influence other innate cells as well as adaptive T and B cells. Notably, a bi-directional interactive network between NKT cells and gut commensal microbiota plays a crucial role in this process. Here, we briefly review recent studies related to the cross-regulation of both NKT cell subsets and how their interactions with other immune cells and parenchymal cells, including hepatocytes and enterocytes, control inflammatory diseases in the liver, such as alcoholic and non-alcoholic steatohepatitis, as well as inflammation in the gut. Overwhelming experimental data suggest that while iNKT cells are pathogenic, type II NKT cells are protective in the liver. Since CD1d-dependent pathways are highly conserved from mice to humans, a detailed cellular and molecular understanding of these immune regulatory pathways will have major implications for the development of novel therapeutics against inflammatory diseases of liver and gut.

## Introduction

The liver is at the center of the interactions between the gut and the rest of the body and little is known about how cellular and molecular interactions in the gut-liver immune axis maintain homeostasis. On the one hand, through the portal circulation, the liver is the primary recipient of gut-derived metabolites and microbial products, and, on the other, the liver secretes products through the biliary system into the gut. In fact, there is a strong association between primary sclerosing cholangitis and inflammatory bowel disease (1, 2). Several factors, including dietary components, particularly fat and alcohol, mucosal damage, infections, medications and toxins, can disturb the intestinal barrier, leading to increased permeability and translocation of bacterial products or metabolites across the epithelial barrier into the portal circulation (3). Under inflammatory conditions, the gut-associated lymphatic tissue is stimulated by the increased influx of pathogen/microbe-associated molecular patterns to secrete pro-inflammatory cytokines (TNFα, IL-1, and IL-6), chemokines, and eicosanoids, all of which can reach the liver and stimulate local responses (4). In this pro-inflammatory environment, both liver parenchymal (hepatocytes) and non-parenchymal cells (intrahepatic lymphocytes, Kupffer cells, sinusoidal endothelial cells and hepatic stellate cells) secrete reactive oxygen species that can contribute to liver injury, inflammation and fibrosis. Thus, in the gut-liver microenvironment, multiple immune and non-immune cells form an interacting network to maintain immune tolerance. In this review, we mainly focus on the interactions between natural killer T (NKT) cell subsets and other innate and adaptive T cells in the gut-liver axis in controlling homeostasis and how activation of different subsets of NKT cells is involved in chronic inflammatory diseases.

## iNKT and type II NKT cell subsets

Both liver and gut are enriched in innate immune cells, including resident macrophages, Kupffer cells, dendritic cells (DC), natural killer cells, and unconventional T cells (5, 6). Unconventional T cells are a diverse population, comprising NKT cells, γδ T cells, mucosal associated invariant T (MAIT) cells, and MHC class I^b^-restricted CD8 T cells. NKT cells are innate-like T cells that express antigen receptors and recognize both exogenous and endogenous lipid antigens presented by a class I MHC-like molecule, CD1d. Following antigenic activation, NKT cells are characterized by their ability to rapidly secrete large amounts of chemokines and cytokines, including IFNγ, TNFα, IL-4, IL-13, IL-17, IL-21, IL-22, and granulocyte-macrophage colony-stimulating factor. These factors modulate immune responses triggered by other innate cells and adaptive T and B cells (7–11).

CD1d-restricted NKT cells exist as two main types based on their TCR usage and lipid recognition. Invariant NKT (iNKT) cells express a semi-invariant TCR consisting of TRAV11 TRAJ18 TCR-alpha chains paired with a limited number of TCR-β chains (TRBV13, TRBV29, or TRBV1) in mice or the orthologous TRAV10 TRAJ18 paired with TRBV25 in humans. Most iNKT cells are strongly reactive to the glycosphingolipid α-galactosylceramide (αGalCer) and are abundant in mice, but less frequent in humans (12). Similar to Th cell subsets, iNKT can be divided into subsets that are defined by their transcription factors and/or cytokines secreted, including iNKT1 (T-bet/IFNγ), iNKT2 (Gata-3/IL-4), iNKT10 (IL-10), and iNKT17 (Rorγt/IL-17) (13–15). Recent studies have indicated that iNKT cells can play a protective or a suppressive role in different diseases, such as microbial infections, chronic inflammation, autoimmunity, allergy, and cancer (16–20).

In contrast, type II NKT cells are not reactive to αGalCer, are more abundant than iNKT cells in humans and consist of CD1d-restricted T cells that express a diverse TCR repertoire but not the semi invariant TCR α-chain expressed by iNKT cells (12). Type II NKT cells can also recognize a variety of lipids antigens, including microbial and endogenous glycolipids and phospholipids as well as endogenous hydrophobic peptides (21). Usually, in comparison to the αGalCer/CD1d/TCR interactions, lipid antigens recognized by type II NKT cells, for example, sulfatides or lysophosphatidylcholine (LPC), binds with lower affinity to CD1d molecules and, accordingly, form relatively less stable tetrameric complexes (22–24), whereas αGalCer-loaded CD1d tetramers form stable complexes (25, 26). Consequently, characterization of type II NKT subsets and exploration of their functions have not progressed as rapidly as for iNKT cells. Nevertheless, most studies have so far suggested an immunosuppressive role of antigen-activated type II NKT cells in several experimental models [recently reviewed in (27)].

## Cross-regulation between iNKT and type II NKT cells

**Figure 1:**
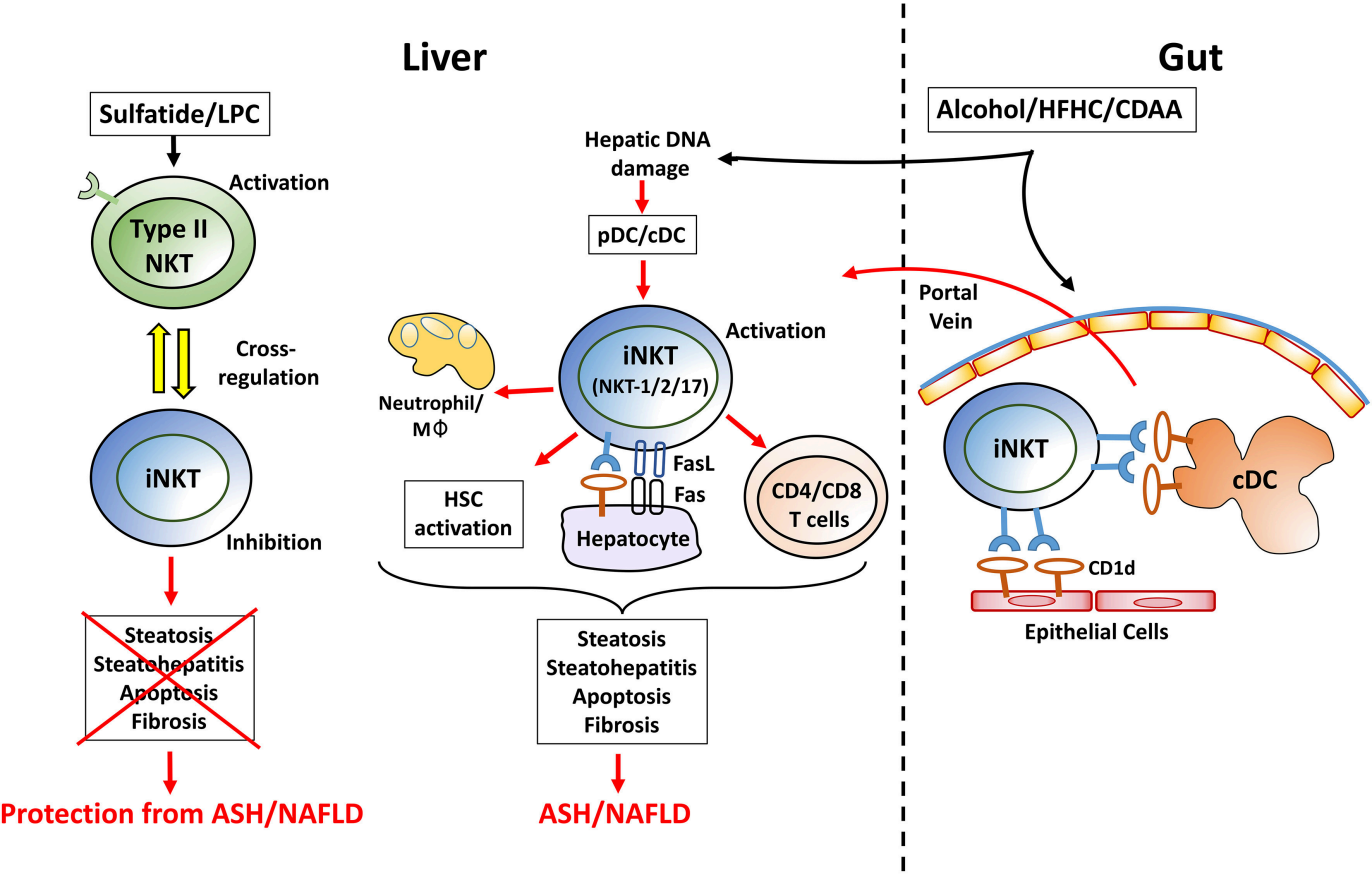
A working model showing a key immune regulatory network mediated by activation of iNKT and/or type II NKT cell subsets in the control of homeostasis in liver and in the gut. **iNKT activation**: In most experimental models of chronic liver diseases, hepatic iNKT cells but not type II NKT cells become rapidly activated. Following feeding of choline-deficient, high fat or alcoholic diets, plasmacytoid DC (pDC) are activated by hepatocyte death and release of mitochondrial DNA. While pDC activate a subset of iNKT cells to secrete IL-17A (iNKT17), conventional DC (cDC) are involved in activation of iNKT secreting IFNγ/IL-4/IL-13 (iNKT1/2). Activation of iNKT cells mediates steatosis and steatohepatitis following recruitment of macrophage/neutrophil and fat deposition in hepatocytes. Furthermore, iNKT cells initiate and accelerate HSC activation and, consequently, fibrosis. This is also associated with increased frequency of migrating cDCs from gut that prime the conventional CD4^+^/CD8^+^ T cells and promote macrophages infiltration into liver leading to enhanced liver damage. Additionally, activated iNKT cells can also express FasL and kill directly Fas-expressing and CD1d^+^ hepatocytes. Similarly, in the gut, following chronic feeding of these diets, the microbiota and lipid profiles are altered resulting in activation of iNKT cells mediated by CD1d^+^ intestinal epithelial cells and/or migratory cDCs. Thus, activated iNKT cells migrate into the liver via portal vein and contribute to liver injury. **Type II NKT activation**: administration of sulfatide and LPC results in activation of hepatic type II NKT cells that cross-regulate iNKT cell-mediated inflammatory cascade and inhibits chronic liver diseases, including ASH and NAFLD. LPC, lysophosphatidylcholine; pDC, plasmacytoid DC; cDC, conventional DC; Mφ, macrophages; HSC, hepatic stellate cells; HFHC, high fat high carbohydrate diet; CDAA, choline deficient amino acid defined diet; ASH, alcoholic steatohepatitis; NAFLD, nonalcoholic fatty liver disease; IBD, inflammatory bowel disease.

We have identified a major subset of murine CD1d-restricted type II NKT cells that can recognize sulfatides as well as LPC and express oligoclonal TCRs with a limited number of Vα- and Vβ-chains (Vα3/Vα1 and Vβ8.1/Vβ3.1) (23, 24, 28, 29). Cross-regulation between iNKT and type II NKT cells has been shown by our laboratory and others in several models of autoimmunity and cancer (8, 11, 30–32). Thus, sulfatide- or LPC-mediated activation of type II NKT cells leads to activation of hepatic plasmacytoid DCs (pDCs) but not conventional DC (cDCs) through a mechanism that depends on IL-12 and macrophage inflammatory protein 2 (MIP2). This results in induction of anergic hepatic iNKT cells unable to secrete cytokines, which is accompanied by tolerization of cDCs and CNS-resident microglia followed by inhibition of the conventional MHC-restricted Th1/Th17 CD4^+^ T cells and protection from autoimmune diseases, such as EAE and diabetes as well as suppression of tumor surveillance (11, 29, 33, 34). Consistently, type II NKT cells mediated induction of anergy in iNKT cells and inhibition of conventional CD4^+^/CD8^+^ T cells prevents immune-mediated liver diseases (24, 27, 30, 33, 35–37) (see Figure 1). In animal models of inflammatory bowel disease, NKT cells can be either protective or pathogenic (38). Interestingly, colonic type II NKT cells appear to play a pathogenic role in the context of increased CD1d expression or iNKT cell deficiency (38–42). Additionally, sulfatide-reactive IL-13Rα2^+^ type II NKT cells are abundant in the lamina propria of ulcerative colitis patients (43) and have recently been shown to be present in human liver (44). How cross-regulation between iNKT and type II NKT cells influences immunity in these compartments during health or disease is an important area of investigation. It was also shown that activation of type II NKT cells by IL-25 prevented high fat diet-induced obesity and transfer of these cells in obese mice improved weight loss and glucose tolerance (11, 31, 45). On the other hand, type II NKT cells can also promote liver inflammation and obesity in animals fed a high-fat diet (46).These different findings indicate that the role of type II NKT cells in the regulation of adipose tissue inflammation and diet-induced obesity remains to be fully understood.

In contrast to a protective role of type II NKT cells, iNKT cells have been shown to promote liver injury contributing to chronic liver diseases, including ischemic reperfusion injury, Concanavalin A-induced hepatitis, Lieber-DeCarli liquid alcohol diet-induced steatohepatitis and diet-induced nonalcoholic fatty liver disease (27, 30, 35, 36, 47–52). Furthermore, we have shown in murine models of chronic alcoholic liver disease (30, 47) as well as in a choline deficient amino acid defined (CDAA) diet-induced nonalcoholic steatohepatitis (NASH) (Maricic et al. submitted manuscript) that direct inhibition of iNKT cells by blocking the RARγ-signaling pathway or by anti-CD1d blocking antibody suppresses disease.

## Choline metabolism and NKT cells involvement in inflammation

Choline deficiency has been implicated in exacerbation of hepatic steatosis and fibrosis in murine models (53, 54), and in nonalcoholic fatty liver disease in humans (55–57). Recent studies have indicated that alterations in the gut microbiota can lead to choline deficiency and contribute to NASH (58, 59). It is notable that the majority of the US population does not meet the daily recommended intake of choline, a situation that is associated with oxidative damage caused by mitochondrial dysfunction and ER stress (55). Furthermore, inflammation in patients with Crohn's disease and ulcerative colitis is also characterized by decreased levels of choline, phosphatidylcholine, and glycerophosphorylcholine due to increased use of choline (60). The presence of trimethylamine-producing bacteria in the gut significantly reduces choline bioavailability and perturbs choline metabolism, which can contribute to NASH (58, 59, 61). In addition, choline and phosphatidylcholine deficiency results in impaired secretion of very low-density lipoproteins and, consequently, accumulation of fat in the liver (steatosis). Interestingly, choline deficiency results in a decrease of colonic type II NKT cells and attenuates dextran sulfate sodium-induced colitis (41). In addition, long-term feeding of a choline-deficient high-fat diet mediate NASH and NASH-induced HCC by promoting liver infiltration by activated iNKT cells and CD8^+^ T cells and induction of inflammatory cytokines (52). These observations suggest an important link between choline metabolism, microbiota, and NKT cells that can control liver inflammation. How activation and cross-regulation of iNKT cells and type II NKT cells are able to control immune responses in NASH and inflammatory bowel disease are currently being investigated in our laboratory.

## NKT cells activation in the liver and gut

In several models of acute and chronic liver inflammation, we have found that hepatic iNKT cells (as determined using αGalCer/CD1d-tetramers) become activated, but not type II NKT cells [reviewed in (31)] (see Table 1). Multiple mechanisms have been shown to be involved in the activation of iNKT cells. Thus, iNKT cells can recognize both microbial and self-derived lipid antigens presented by CD1d-expressing professional APCs, such as DC, resulting in direct activation of iNKT cells. Also, IL-12 and type I IFN produced by activated DC in response to ligands of toll-like receptor (TLR)-4 and TLR-9, respectively, can strengthen NKT activation (62, 63). Furthermore, it has been shown that CD1d-dependent activation of iNKT cells by hepatocytes control liver inflammation (64). Additionally, iNKT cells also become activated by a combination of cytokines, such as IL-12 and IL-18, in the absence of a CD1d-bound agonist (27, 31).

**Table 1:** Differential activation, accumulation and influence of NKT cell subsets on other innate cells in liver inflammation.

**iNKT cells^a^**					**Type II NKT cells^b^**	**Myeloid cells^c^**	**NK cells^d^**
**Liver**	**Tetramer^+^**	**Cytokines^e^**	**FasL**	**Apoptosis^f^**			
IRI	NC	IFNγ	NC	NC	ND	↑↑	↑
Con A	↓	IFNγ, IL-4	↑↑	↑↑	ND	↑↑	↑
Alcohol C + B	↑↑	IFNγ	NC	NC	NC	↑↑	NC
Alcohol Chronic	NC/↓	IFNγ	↑	↑	↑↑	↑↑	NC
CDAA diet	↓	IL-17, IFNγ, IL-4, IL-13	ND	ND	ND	↑↑	↑

a *iNKT cells were analyzed in liver mononuclear cells using αGalCer-loaded CD1d tetramers and identified as double positive cells for αGalCer/CD1d tetramer and TCRβ (αGalCer/CD1d tetramer^+^TCRβ^+^) in the NK1.1^+^ gate*.

b *Type II NKT cells were also analyzed in liver mononuclear cells and defined as double positive cells for TCRβ and NK1.1 (TCRβ^+^NK1.1^+^) in the αGalCer/CD1d tetramer^−^ gate or sulfatide/CD1d-tetramer^+^ cells*.

c *Myeloid cells included both monocytes, neutrophils and macrophages*.

d *NK cells were defined as NK1.1^+^TCRβ^−^ cells*.

e *Cytokines secretion was determined in culture supernatants after stimulation with PMA & Ionomycin by FACS using BD Cytometric bead array*.

f *Apoptotic cells were identified as Annexin V^+^ cells*.

*IRI, Ischemia Reperfusion Injury; Con A, Concanavalin A-induced hepatitis; Alcohol C+B, chronic feeding of Lieber-DeCarli liquid diet for 10 days plus binge on day 11; Alcohol Chronic, chronic feeding of Liber deCarli liquid alcohol diet for 6–8 weeks; CDAA diet, chronic feeding of Choline deficient amino acid defined diet for 20 weeks. NC, not changed; ND, not determined*.

Since NKT cells are also localized in the intestine and CD1d is also expressed by intestinal B cells, DC, macrophages as well as intestinal epithelial cells, they can also mediate lipid-activation of NKT cells thus participating in the intestinal homeostasis and regulation of intestinal immunity. In fact, CD1d-dependent colonization with specific commensal gut bacteria has been shown to be crucial in the maintenance of mucosal homeostasis (65, 66). More recently, the importance of CD1d-dependent activation of iNKT cells in the gut by intestinal lipids presented by CD11c^+^ DC has been shown for control of intestinal homeostasis (64, 67). Additionally, it has been also shown that altered commensal gut bacteria induce hepatic accumulation of CXCR6^+^ iNKT cells that are able to inhibit liver cancer (68). Furthermore, it is well-known that migratory CD103^+^ DC in the gut, which also express high levels of CD1d, play an important role in the activation of iNKT cells (69, 70).

Based upon data from other laboratories and ours, we propose the following model of how diet can lead to activation of iNKT cells in the gut and liver (Figure 1). Also, how sulfatide- or LPC-mediated activation of type II NKT cells inhibits iNKT-mediated inflammatory cascade followed by protection from chronic inflammatory liver diseases. Alcoholic, choline-deficient, or high-fat diets can initially damage hepatocytes, leading to release of mitochondrial DNA and accumulation and activation of pDCs in the liver. The pDCs activate IL-17-secreting iNKT cells (iNKT17), which induces IL-17 signaling leading eventually to the expansion of conventional Th17 T cells. Chronic feeding of these diets leads to further changes in the gut microbiota, resulting in enterocytes or cDC-mediated presentation of lipids to iNKT cells. Additionally, migration of the CD103^+^CD11b^−^ cDC and activated iNKT cells into liver results in activation or expansion of both MHC-restricted CD4^+^/CD8^+^ T cells and IFNγ/IL-13-secreting iNKT cells as well as hepatic stellate cell activation and fibrosis. Collectively, these data suggest that DC subsets as well as CD1d-expressing parenchymal cells are involved in activation of iNKT cells in both liver and gut.

## Bimodal interactions between NKT cells and gut microbiota

Mucosal barrier in the gut plays a crucial role in keeping gut commensal microbes away from the host immune system. Accordingly, in the DSS-induced colitis model, live bacteria have been found in the liver at increased numbers (71). Similarly, high levels of bacterial colonization have been shown in portal blood, liver and peritoneum of patients with Crohn's disease (72). In different settings, the interactions in the gut-liver axis have been shown to be critical in the pathogenesis of several inflammatory liver diseases (4, 73, 74). NKT cells contribute to immunosurveillance in the small and large intestine. Thus, the frequency as well as the functional maturation of NKT cells in the lamina propria and gut epithelium is controlled by lipids derived from the commensal microbiota. At the same time, CD1d activation of NKT cells in the gut can modulate neonatal colonization with commensal bacteria (75, 76). Interestingly, bacterial lipids related to αGalCer can be synthesized by Bacteriodes species and can activate or inhibit iNKT cells (77, 78). Therefore, the frequency, maturation and phenotype of iNKT cells is greatly impacted by the gut microbiota in mice. It is not clear yet whether, in humans, commensal microbes can have a similar effect on iNKT cells. Similarly, it has recently been shown that NKT cells can control microbial communities in the gut. In the absence of NKT cells, mice develop much more severe colitis (66). In addition, lipid presentation within the gut by CD1d-expressing CD11c^+^ DCs and macrophages induces activation of intestinal iNKT cells controlling the intestinal bacteria composition (67). Similarly, CD1d-mediated lipid presentation by hepatocytes controls homeostasis of hepatic iNKT cells. Altered CD1d-dependent presentation results in increased hepatic iNKT cell numbers and hepatic inflammation (64). Therefore, experimental data in murine models suggest reciprocal interactions between NKT cells and gut bacteria, and that these interactions have implications in targeting NKT cells or the microbiota in chronic liver diseases or inflammatory bowel disease. Whether similar reciprocal interactions may occur in human need to be determined. It has been shown that gut bacteria derived from chronic alcoholic hepatitis patients can induce liver disease in mice upon colonization (79), and that intestinal microbiota manipulation prevents alcohol-induced liver injury (80). Since murine alcoholic liver disease is mediated by iNKT cells, it is likely that products derived from critical microbes or diets can activate iNKT cells. Collectively, these studies indicate an important multimodal interaction among gut microbes, NKT cell subsets, and both immune and parenchymal cells in the maintenance of gut and liver homeostasis.

## Future perspective

The studies reviewed here have revealed a central role of NKT cell subsets in the maintenance of immune homeostasis in the liver and gut. Detailed knowledge of the cellular and molecular interactions among both NKT cell subsets, other innate and adaptive lymphocytes, and the microbiota will be key for developing novel intervention strategies for chronic inflammatory diseases, such as alcoholic liver disease, nonalcoholic steatohepatitis and inflammatory bowel disease. It is becoming increasingly clear that iNKT cells contribute to inflammation while type II NKT cells are protective (30), a clear understanding of the role of these unconventional T cells in large cohorts of patients with chronic inflammatory diseases of liver and gut is needed to develop inhibiting and activating agonists of iNKT cells and type II NKT cells, respectively, as novel therapeutic strategies for these diseases.

## Author contributions

All authors listed have made a substantial, direct and intellectual contribution to the work, and approved it for publication.

### Conflict of interest statement

VK is a Scientific Co-founder and Consultant for the GRI-Bio, La Jolla, California. The remaining authors declare that the research was conducted in the absence of any commercial or financial relationships that could be construed as a potential conflict of interest.
